# Supplementary material for: The Influence of Prone Positioning on Energy and Protein Delivery in COVID-19 Patients Requiring ECMO Support
Source: Nutrients. 2024 Oct 18;16(20):3534. doi: 10.3390/nu16203534 (PMC11510455; doi:10.3390/nu16203534)
Supplement: Supplementary file 1 [file nutrients-16-03534-s001.zip › nutrients-3236848-supplementary.pdf]

Table S1. Data on medical nutrition therapy in kcal and gram

Nutrition data	Overall	Days in supine	Days in prone	p-value
Total calories (kcal), mean (Std.)	1485 (560.3)	1491.5 (565.1)	1461.8 (544.2)	**0.022**
Calories (kcal) EN, mean (Std.)	1121.8 (640.9)	1191.6 (634.3)	850.3 (592.8)	**<0.001**
Calories (kcal) PN, mean (Std.)	861.4 (452.2)	791.8 (436.5)	999.4 (452.1)	**<0.001**
Calories (kcal) propofol, mean (Std.)	230.2 (129.5)	205.9 (127.3)	303 (106.8)	**<0.001**
Carbs (g), mean (Std.)	162.3 (72.9)	165.5 (72.2)	151.4 (74.2)	**<0.001**
Carbs (g) EN, mean (Std.)	137.6 (79.5)	145.9 (77.8)	105.2 (78.2)	**<0.001**
Carbs (g) PN, mean (Std.)	109.7 (69.1)	100.7 (67.0)	127.4 (69.9)	**<0.001**
Protein (g), mean (Std.)	62.0 (30.0)	61.8 (29.2)	62.7 (32.8)	0.912
Protein (g) EN, mean (Std.)	46.9 (29.3)	50.0 (29.4)	34.8 (25.5)	**<0.001**
Protein (g) PN, mean (Std.)	54.9 (33.6)	49.4 (32.1)	66.5 (33.7)	**<0.001**
Fat (g), mean (Std.)	59.2 (25.8)	59.1 (25.6)	59.8 (26.5)	0.578
Fat (g) EN, mean (Std.)	40.2 (24.3)	42.9 (24.0)	30.3 (22.9)	**<0.001**
Fat (g) PN, mean (Std.)	19.6 (17.7)	18.1 (16.4)	22.4 (19.8)	0.076
Fat (g) propofol, mean (Std.)	20.9 (11.8)	18.7 (11.6)	27.5 (9.7)	**<0.001**

Abbreviations: EN, enteral nutrition; g, gram; kcal, kilocalories; PN, parenteral nutrition; Std., standard deviation; %, percent.

Table S2. Data on mean daily protein delivery (g/kg BW/d)

Nutrition data	Overall	Days in supine	Days in prone	p-value
All ECMO days				
Overall, n (%) | mean (Std.)	2344 (100) | 0.70 (0.4)	1830 (78.1) | 0.70 (0.3)	514 (21.9) | 0.68 (0.4)	**0.007**
EN, mean (Std.)	0.49 (0.4)	0.54 (0.4)	0,32 (0.3)	**< 0.001**
PN, mean (Std.)	0.21 (0.4)	0.17 (0.3)	0.36 (0.5)	**< 0.001**
ECMO day 1-3				
Overall, n (%) | mean (Std.)	305 (100) | 0.62 (0,39)	183 (60) | 0.59 (0.37)	122 (40) | 0.65 (0.41)	0.287
EN, mean (Std.)	0.27 (0.29)	0.32 (0.31)	0.19 (0.24)	**< 0.001**
PN, mean (Std.)	0.35 (0.42)	0.27 (0.37)	0.46 (0.47)	**< 0.001**
ECMO day 1-7				
Overall, n (%) | mean (Std.)	693 (100) | 0.65 (0.37)	433 (62.48) | 0.64 (0.37)	260 (37.52) | 0.68 (0.37)	0.341
EN, mean (Std.)	0.32 (0.31)	0.36 (0.32)	0.25 (0.27)	**< 0.001**
PN, mean (Std.)	0.33 (0.42)	0.28 (0.40)	0.43 (0.44)	**< 0.001**
ECMO day 8-14				
Overall, n (%) | mean (Std.)	548 (100) | 0.73 (0.35)	428 (78.10) | 0.73 (0.34)	120 (21.90) | 0.75 (0.38)	0.733
EN, mean (Std.)	0.48 (0.36)	0.50 (0.37)	0.42 (0.32)	0.072
PN, mean (Std.)	0.25 (0.40)	0.23 (0.36)	0.33 (0.50)	0.308
ECMO day 15-30				
Overall, n (%) | mean (Std.)	693 (100) | 74.08 (29.72)	580 (83.69) | 74.76 (29.99)	113 (16.31) | 70.55 (28.20)	0.072
EN, mean (Std.)	0.55 (0.33)	0.59 (0.33)	0.34 (0.26)	**< 0.001**
PN, mean (Std.)	0.14 (0.29)	0.11 (0.26)	0.30 (0.40)	**< 0.001**

Abbreviations: BW, body weight; d, day; ECMO, extracorporeal membrane oxygenation; EN, enteral nutrition; n, number; g, gram; kg, kilogram; PN, parenteral nutrition; Std., standard deviation; %, percent.

Table S3. Patient-specific daily calorie and protein intake

	Daily calorie del. (% of requ.) overall, mean (Std.)	Daily calorie del. (% of requ.) in supine, mean (Std.)	Daily calorie del. (% of requ.) in prone, mean (Std.)	p-value	Daily protein del. (g/kg BW/d) overall, mean (Std.)	Daily protein del. (g/kg BW/d) in supine, mean (Std.)	Daily protein del. (g/kg BW/d) in prone, mean (Std.)	p-value
Group A	65.3 (21.0)*	65.3 (21.0)**	/	/	0.63 (0.27)⁺	0.63 (0.27)⁺⁺	/	/
Group B	69.8 (17.8)*	69.2 (20.3)**	65.7 (21.1)	0.159	0.66 (0.22)⁺	0.65 (0.24)⁺⁺	0.64 (0.29)	0.788

* p-value for overall daily calorie del. compared in group A vs. group B; p = 0.135
** p-value for daily calorie del. in supine compared in group A vs. group B; p = 0.294
⁺ p-value for overall daily protein del. compared in group A vs. group B; p = 0.872
⁺⁺ p-value for daily protein del. in supine compared in group A vs. group B; p = 0.545
Abbreviations: BW, body weight; d, day; del., delivery; g, gram; kg, kilogram; requ., requirements; Std., standard deviation; %, percent.
